# Inflammation of the male reproductive system: clinical aspects and mechanisms

**DOI:** 10.3389/fendo.2025.1547020

**Published:** 2025-06-18

**Authors:** Binghao Bao, Haolang Wen, Fei Wang, Daishu Han, Baoxing Liu

**Affiliations:** ^1^ Department of Andrology, China-Japan Friendship Hospital, Beijing, China; ^2^ Graduate School, Beijing University of Chinese Medicine, Beijing, China; ^3^ Institute of Basic Medical Sciences, Chinese Academy of Medical Sciences, School of Basic Medicine, Peking Union Medical College, Beijing, China

**Keywords:** male reproductive system, inflammation, spermatozoa, diagnosis, treatment

## Abstract

The male reproductive system (MRS) is composed of multiple highly organized components that are relatively separated and adopt special histological and immunological microenvironments. The inflammatory conditions are most frequent pathological states of the MRS. Retrograde microbial infections are pivotal etiological factors contributing to acute inflammation in the MRS. However, the majority of inflammatory conditions in the MRS can be caused by non-infectious factors and manifest chronic inflammation, which may significantly affect quality of life and fertility. The mechanisms underlying the non-infectious inflammation in the MRS remain largely elusive. Therefore the diagnosis and treatment of the non-infectious inflammation in the MRS are confusing. Recent studies demonstrated that immunogenic spermatozoa could be potential triggers of the non-infectious inflammation in the MRS. This review article outlines current status of the diagnosis and the treatment of inflammation and underlying mechanisms in the MRS. We also highlight the prioritized issues for future studies.

## Introduction

**Figure 1:**
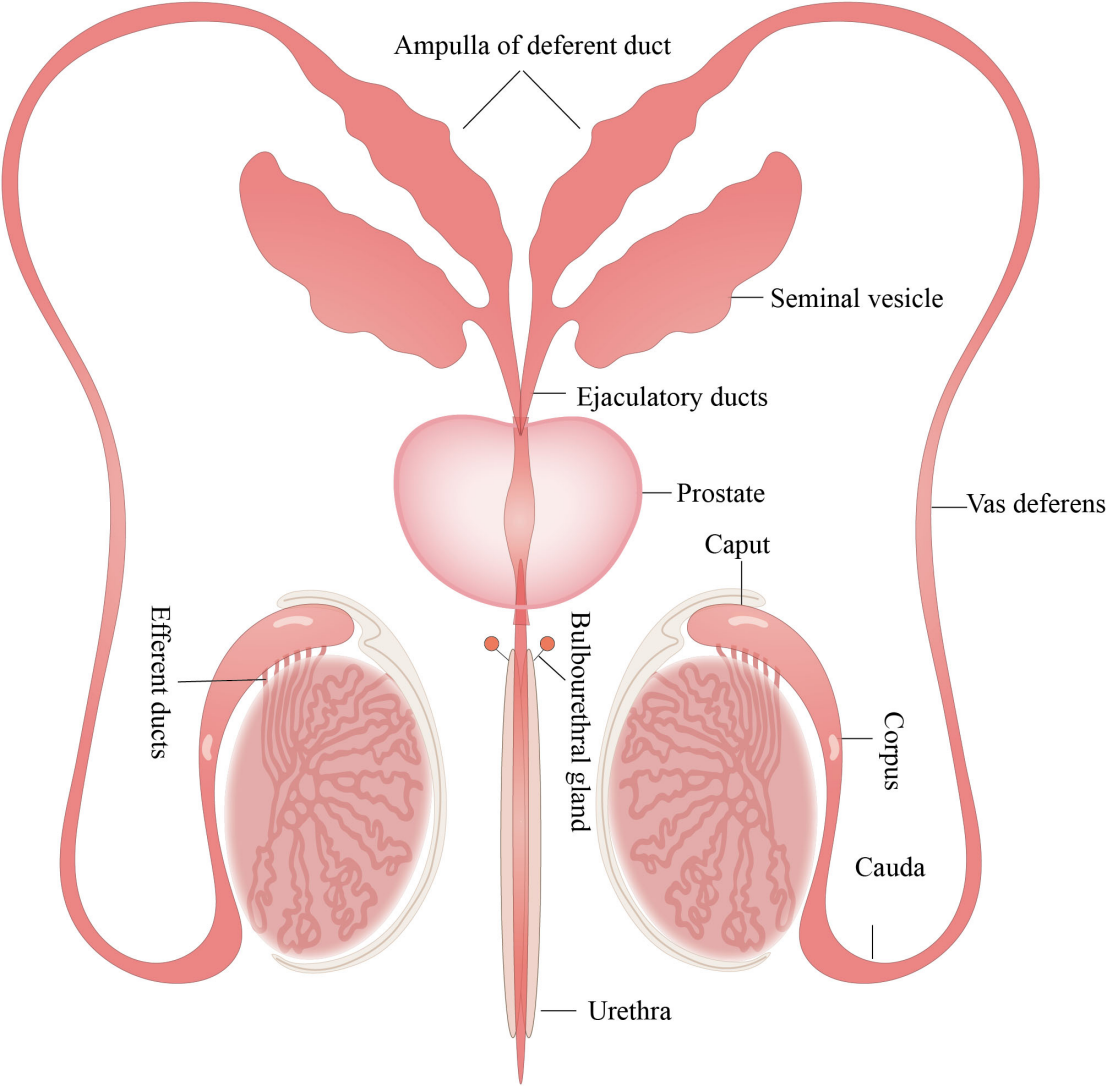
Structure of the MRS. The MRS consists of the testes, epididymis, prostate gland, seminal vesicles, and bulbourethral glands. These organs are connected by reproductive ducts: the vas deferens, ampulla of deferent duct, ejaculatory ducts and urethra. All figures were drawn using Adobe Illustrator software.

The male reproductive system (MRS) comprises internal and external genitalia, each with its distinct anatomic location and enclosed tissue microenvironment (1). The internal genitalia encompass the gonads (testes), reproductive ducts (epididymis, vas deferens, ejaculatory duct, and urethra), and accessory glands (seminal vesicles (SVs), prostate, and bulbourethral glands). The external genitalia include the scrotum and penis (**Figure 1**).

Inflammatory conditions frequently occur in the MRS and may significantly impair quality of life and male fertility (2). MRS impairs fertility through distinct pathways. In prostatitis, E. coli LPS-TLR4 signaling similarly elevates these cytokines. IL-6 triggers neutrophil-derived hypochlorous acid damaging sperm membranes, whereas IL-8 sustains inflammation via leukocyte recruitment, collectively compromising fertility (3, 4). Viral infections compromise male fertility by invading the testes, inducing systemic inflammation that disrupts hormones, and generating oxidative stress, which causes DNA damage and impairs sperm motility (5). While retrograde microbial infections are important etiologic factors (6, 7), many cases of chronic inflammation in the MRS lack evidence of microbial infection and are termed non-infectious inflammation. The diagnosis of acute inflammation triggered by microbial infection is relatively straightforward. However, the etiologic factors and pathomechanisms underlying non-infectious inflammation in the MRS are largely unclear, thereby complicating accurate diagnosis and targeted treatment.

Lifestyle and environmental factors play crucial roles in the development of non-infectious inflammation in the MRS. Lifestyle factors such as diet, exercise, and stress, alongside environmental exposures to toxins, have been identified as key contributors to inflammatory processes. Poor lifestyle habits leading to obesity can induce inflammation through multiple pathways. Obesity, resulting from poor dietary and physical activity patterns, directly elevates proinflammatory cytokine levels, which in turn may impair reproductive health (8). Toxins such as TCDD (2,3,7,8-tetrachlorodibenzo-p-dioxin, commonly known as dioxin) can suppress Klotho expression in Sertoli cells, activate the p65/NLRP3 pathway, and promote the release of inflammatory cytokines, thereby aggravating inflammatory damage (9). Similarly, diazinon has been reported to activate NF-κB and MAPK pathways, triggering testicular inflammation and increasing the production of proinflammatory factors such as TNF-α, IL-6, COX-2, and iNOS. This process compromises the integrity of the blood-testis barrier, permitting the invasion of immune cells and toxins into seminiferous tubules, further promoting reproductive cell apoptosis (10).

While inflammatory conditions occur in most all organs of the MRS, the prevalence and etiologic factors vary in individual organs. The frequency of inflammation is relatively high in the urethra and prostate compared to SVS, vas deferens, epididymis and testis. Inflammatory conditions in the urethritis, are primarily induced by microbial infections. In contrast, most cases of inflammation in major genital gonads and accessory glands, including the prostate, SV, epididymis, and testis, are caused by non-infectious etiologic factors (11, 12). The inflammatory characteristics of individual organs are associated with their anatomical location, tissue structure, and the immunological microenvironment (13). This article reviews our understanding of mechanism and clinical applications of the inflammatory conditions in major organs of the MRS, including the testis, epididymis, seminal vesicle, and prostate, because the inflammations in these organs can seriously impact our quality of life and male fertility.

## Orchitis

### Tissue structure and immune environment of the testis

**Figure 2:**
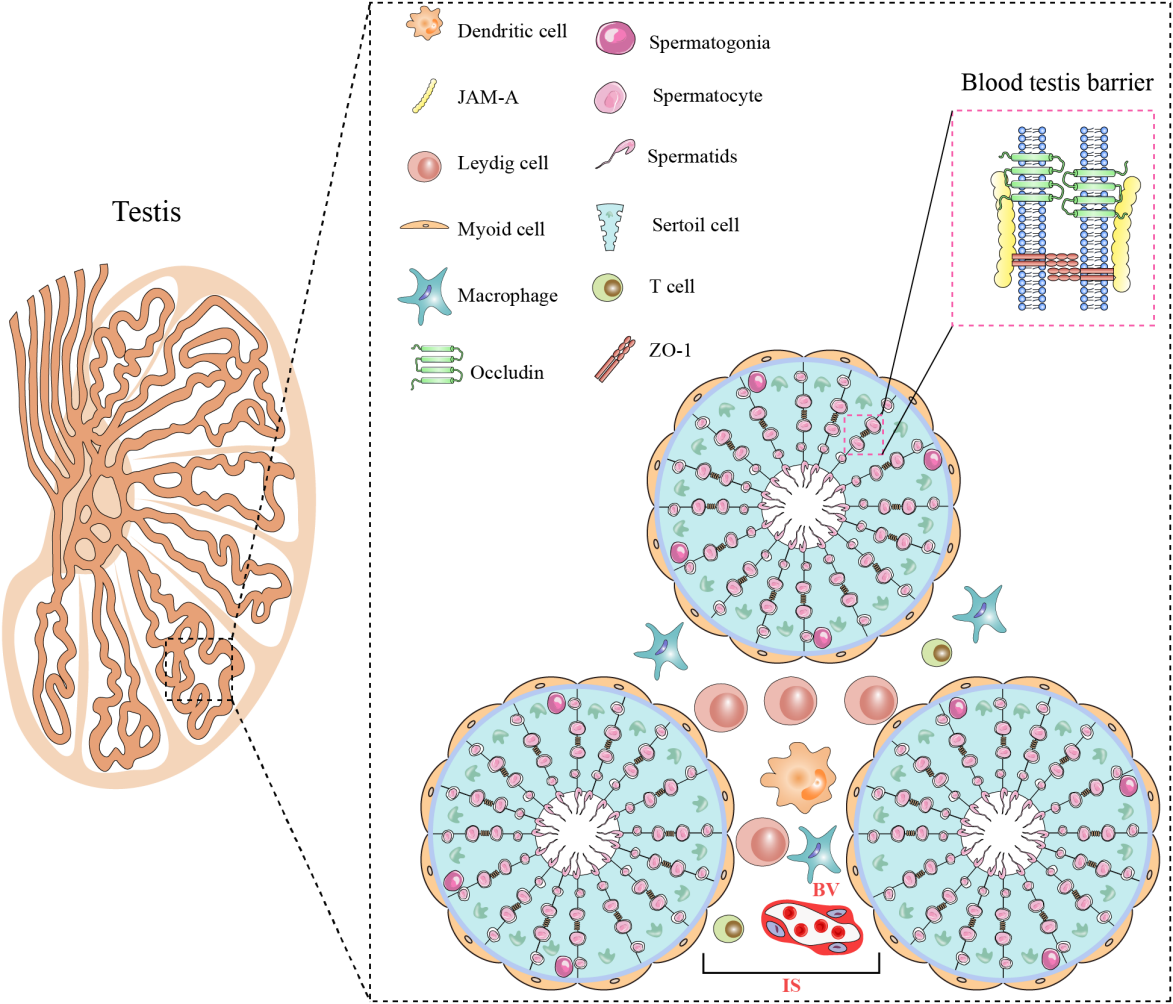
Histological structure and cellular components of the testis. The testis consists of two main regions: the seminiferous tubule (ST) and the interstitial space (IS). Myoid cells surround the ST. Spermatogenesis occurs within the seminiferous epithelium, composed of Sertoli cells and spermatogenic cells at various stages of development. Adjacent Sertoli cells are interconnected through various cellular junctions, creating the BTB. The IS houses Leydig cells various immune cells including macrophages, dendritic cells, and T cells, as well as blood vessels (BVs). All figures were drawn using Adobe Illustrator software.

The testis possesses a special histologic structure and microenvironment for conducting its functions, primarily spermatogenesis and testosterone production, and consists of seminiferous tubules and interstitial space (**Figure 2**). The wall of seminiferous tubules are primarily composed of peritubular myoid cells with contractile functions. Sertoli cells in the wall of the seminiferous epithelium tightly embrace germ cells at various stages of development and support spermatogenesis. The interstitial space consists of Leydig cells, immune cells, blood vessels, and lymphatic vessel (14). Leydig cells synthesize testosterone (**Figure 2**).

The testis is a remarkable immune-privileged organ that is regulated by tissue structure, local immune suppression, and systemic immune tolerance (15). The blood-testis barrier (BTB) has been considered to play an important role in maintaining testicular immune-privileged status. The BTB is formed by adjacent Sertoli cells through various connections, including tight junctions, adherens junctions, and gap junctions. The BTB efficiently isolates the tubular lumen from the interstitial space, preventing immune molecules in the interstitial space from entering the testicular lumen, thereby protecting germ cells from autoimmune reactions (16). In addition to the BTB, testis immune privilege is also due to controlled antigen presentation and immune regulation (17).

To maintain its immune-privileged environment, the testis relies on an effective local innate immune defense system involving interstitial immune cells and tissue-specific cells. Macrophages are the predominant immune cell type in the testicular interstitium, comprising approximately 80% of testicular immune cells, and serve as the first line of defense against microbial infections. In addition, tissue-specific cells of the testis—including Leydig cells, Sertoli cells, and germ cells—also possess innate defensive capabilities (15). These testis-specific cells express a variety of pattern recognition receptors (PRRs), and activation of these PRRs helps combat microbial infections. However, these defenses do not render the testis immune to inflammation; orchitis is one such inflammatory condition in the male reproductive system (13).

### Characterization of orchitis

Isolated orchitis is relatively rare and usually presents concomitantly with epididymitis. Therefore, the precise incidence of orchitis remains unclear. Mumps virus (MuV) is a major cause of most isolated orchitis cases, with approximately 18% of mumps patients exhibiting symptoms of orchitis (18). Apart from hemodiffusion of viruses, genitourinary infections do not primarily affect the testis. Orchitis typically occurs with concurrent epididymitis due to infection by similar causative pathogens. Hematogenous spread is the primary route for isolated testicular infections (19, 20), whereas orchitis is mainly a result of the inflammatory process within the epididymitis after retrograde infection via the genital tracts (21, 22).

### Pathomechanisms

The etiology of orchitis primarily encompasses microbial infections (viruses, bacteria, fungi, and Mycobacterium tuberculosis), chemical toxicants (amiodarone and mercury compounds), and physical injuries (e.g., genital trauma and vasectomy) (6). Hematogenous spread of viruses is a significant cause of orchitis, and MuV is a typical virus that leads to orchitis. MuV-induced orchitis suppresses testosterone synthesis, induces germ cell apoptosis, and impairs BTB integrity, in which high levels of tumor necrosis factor-alpha (TNF-α) play a crucial role (23–25). In addition to viral infections, chemical toxicants and physical injuries can also disrupt testicular immune homeostasis. Amiodarone accumulation in the testes triggers oxidative stress and TNF-α/IL-6-mediated inflammation (26), while cadmium compromises BTB integrity, promoting immune cell infiltration and germ cell damage (27). Similarly, testicular trauma and ischemia-reperfusion injury activate the NLRP3 inflammasome, amplifying inflammation through IL-1β and NF-κB pathways (28). Moreover, vasectomy-induced spermatozoa leakage may lead to autoimmune orchitis, characterized by macrophage activation and chronic inflammatory responses (29). These non-infectious factors further exacerbate inflammatory damage in the male reproductive system, highlighting the multifaceted nature of orchitis pathogenesis.

Immune-related factors potentially contribute to chronic orchitis. Specifically, autoimmune orchitis arises from autoimmune responses that result in the generation of testicular-specific autoantibodies (30). and this can subsequently constitute a potential factor in the decline of male fertility. Nonetheless, the precise pathologic mechanisms underlying this action remain elusive. Studies conducted on animal models of experimental autoimmune orchitis have revealed the disease’s hallmark features: immune cell infiltration (primarily macrophages, dendritic cells, and T cells) into the stroma, autoantibody production against testicular antigens, secretion of pro-inflammatory mediators, and disorders in steroidogenesis (31–33). Pro-inflammatory cytokines such as TNF-α, NO, and interleukin-6 (IL6) that are synthesized by testicular macrophages have the potential to impair spermatogenesis. This impairment has been attributed to the induction of spermatogenic cell apoptosis and dysfunction in Sertoli cells, with Toll-like receptor 2 (TLR2) and TLR4 being pivotal in this process (34).

These cytokines are primarily secreted by activated macrophages and dendritic cells upon recognition of pathogen-associated molecular patterns (PAMPs) and damage-associated molecular patterns (DAMPs) via Toll-like receptor (TLR)-mediated signaling pathways (35). In contrast, IL-10 serves a critical immunoregulatory function by suppressing excessive inflammation and promoting tissue repair through the modulation of macrophage polarization. IFN-γ, predominantly produced by Th1 cells and natural killer (NK) cells, contributes to the activation of antigen-presenting cells and the maintenance of chronic inflammatory responses in persistent infections (36).

### Treatment of orchitis

Treatment of orchitis is primarily supportive, including bed rest and local cooling to alleviate pain. The initial treatment for acute orchitis consists of antibiotics against the most likely pathogens based on the patient’s sexual history and demographics. Antibacterial drugs are primarily effective for bacterial orchitis, but are unsuitable for treating viral orchitis. Once the pathogen is identified, it is essential to adjust the treatment according to drug-sensitivity results; and most patients achieve successful therapeutic outcomes (37). However, antibiotic resistance is a concern, especially in cases of recurrent infection. The choice of antibiotics should be based on the results of pathogen-specific testing to avoid overuse of broad-spectrum antibiotics. For viral orchitis, antibiotics are ineffective (38). Most MuV-induced orchitis cases resolve spontaneously within 3–10 days, requiring supportive care only. Nevertheless, it is important to recognize that viral orchitis may cause long-term complications (39), such as reduced testosterone production or infertility, which are not addressed by the standard treatment.

Epidemiologic and pathologic data for chronic orchitis or asymptomatic orchitis are lacking. If chronic bacterial orchitis or granulomatous orchitis is caused by infection, treatment usually consists of antibiotics against the identified pathogens. For sexually transmitted infections, partners must also be examined and treated concurrently. For those patients with persistent inflammatory conditions after appropriate antibiotic treatment, a combination of antibiotics and non-steroidal anti-inflammatory drugs is recommended (40). However, the specific efficacy of anti-inflammatory treatments for chronic orchitis remains uncertain, and there is no established protocol for immunosuppressive treatment of chronic orchitis using corticosteroids. The use of corticosteroids or other immunosuppressive agents lacks robust evidence in the context of orchitis, and further studies are needed to establish their efficacy and safety. These treatments may pose risks, including immunosuppression, increased susceptibility to infections, and potential long-term side effects such as osteoporosis and metabolic disturbances (41). The challenges in the treatment of chronic orchitis are due to an insufficient understanding of etiologic factors and underlying pathophysiology, which are worthy of future investigation.

## Epididymitis

### Histologic structure and immune environment of the epididymis

**Figure 3:**
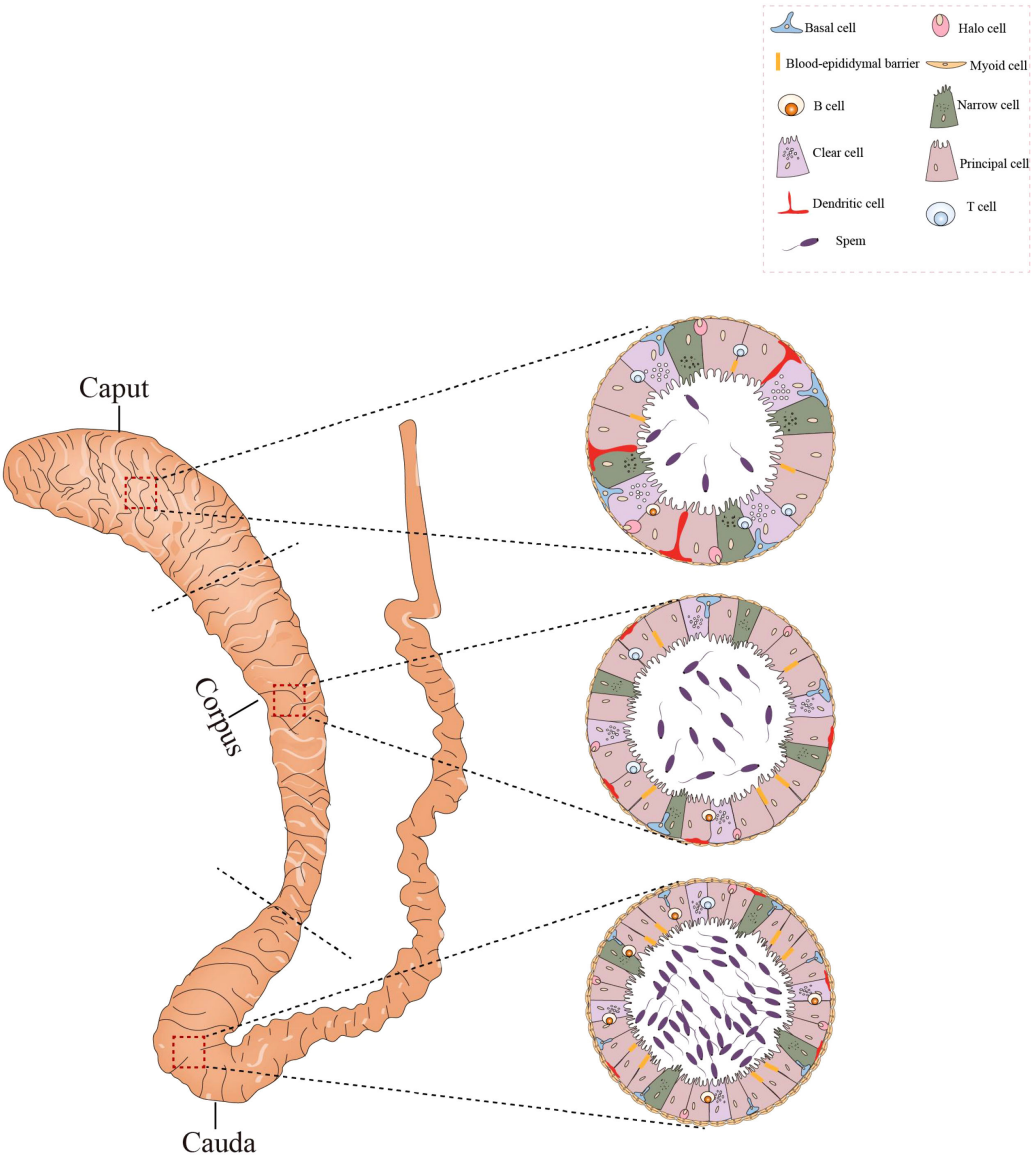
Histological structure and cellular components of the epididymis. The epididymis consists of three sections: the caput, corpus, and cauda. Its epithelium harbors diverse cell types, such as basal cells, lymphocytes, clear cells, dendritic cells, and macrophages. In the caput, dendritic cells can extend towards the tight junctions between epithelial cells. The caput contains a higher number of T cells compared to the cauda, whereas the concentration of B cells is greater in the cauda. All figures were drawn using Adobe Illustrator software.

The epididymis can be divided into three segments: caput, corpus, and cauda (**Figure 3**). The caput connects the testis via efferent ductules, and the cauda is linked to the vas deferens. The epididymis consists of the epididymal tubule and interstitium. The epididymal tubule is composed of pseudostratified epithelium containing diverse epithelial cells (42). Various immune cells, predominantly macrophages and dendritic cells, are abundant within the epididymis. The macrophages are localized in the interstitium and peritubular areas, whereas dendritic cells are positioned on the bases of the epididymal tubule (**Figure 3**).

Although immune cells can be found in the caput, corpus, and cauda regions of the epididymis, their numbers decrease from the caput to cauda epididymis. The distribution of immune cells may, then, play a role in inhibiting the transmission of pathogens from the epididymis to the testis (43). Since sperm are immunogenic, the epididymis must possess a specialized environment to avoid an autoimmune response during the storage of sperm in the epididymis. The immune cells in the epididymis may also play a role in maintaining immune tolerance for the sperm.

The epithelium of the epididymal tubule forms a barrier of tight junctions between neighboring epithelial cells, namely, the blood-epididymis barrier (BEB). The epididymis exhibits greater susceptibility to inflammatory and autoimmune reactions compared to the test is due to the less effective BEB relative to the BTB, compounded by the leaky junctions in the efferent ducts, which further contribute to heightened immune responses—particularly in the caput region. The epididymis possesses its own defense system. For example, the epididymis secretes many defensins with antimicrobial activity (44); and the epithelial cells of the epididymis can express various PRRs, initiating innate immune responses (45, 46).

### Epidemiology

Epididymitis is a common disease in the MRS (19). Approximately 600,000 cases are reported annually in the United States, with the majority of cases occurring in men aged 18–35 years (47). In a U.S. Army study, the highest incidence was observed in men aged 20–29 years (20). Epididymitis is more common than orchitis and is a prevalent condition in men aged 18–50 years (48).

Chronic epididymitis, in particular, can severely impair fertility (49). Prolonged chronic epididymitis can damage the structure of the epididymal tubule, leading to obstructive azoospermia (49), and the outcome of treatments for chronic epididymitis is generally unpredictable (50).

In male infertility patients, chronic non-specific epididymal inflammation, as well as the sequelae of acute, recurrent, or chronic genital inflammation, is commonplace. Up to 35% of patients with subfertility exhibit inflammation in the genital tract (51). While clinical and pathologic studies have established a connection between epididymitis and infertility (52, 53), additional research on the underlying mechanisms is required.

### Pathologic mechanism

Epididymitis can be classified as acute or chronic. In acute epididymitis, symptoms persist for less than 6 weeks and are characterized by pain and swelling of the epididymis. Chronic epididymitis is characterized by pain, typically without swelling, and a duration of greater than 3 months (54). Epididymitis is the most common infectious disease within the scrotum, and retrograde infection is the primary cause of epididymitis. Chemical irritation caused by urine reflux and bacterial infections are common causes of epididymitis (55).

The causative bacteria of acute epididymitis are correlated with patient age. Chlamydia trachomatis and Neisseria gonorrhoeae are the most prevalent causes among sexually active males aged 14–35 years. In individuals older than 35 years, acute epididymitis is predominantly caused by Escherichia coli (E. coli). Prostatic hyperplasia may lead to bladder outlet obstruction and subsequent urine reflux into the vas deferens and is also an etiologic factor in epididymitis (56, 57), with non-infectious epididymitis the most commonly observed. Non-infectious acute epididymitis can arise from an adverse drug reaction, bladder outlet obstruction, or underlying systemic conditions such as granulomatous disease or Behçet’s syndrome, which cause urine reflux into the vas deferens (58).

### Diagnostic and therapeutic approaches

#### Acute epididymitis

Acute epididymitis often presents with intensifying pain and swelling in the posterior scrotum for 1–2 days, and symptoms may be accompanied by fever, hematuria, dysuria, and an increased frequency of urination (59). A detailed medical history and physical examination are essential for the diagnosis of epididymitis, and urinalysis and urine culture are performed to evaluate for infectious microorganisms. All patients with an abnormal screening urinalysis should be subjected to a urine culture. Patients under age 35 years who are sexually active or those who frequently change sexual partners should undergo nucleic acid amplification tests to detect gonorrhea and chlamydia infections (60).

A color Doppler ultrasonographic examination is necessary for patients suspected of epididymitis to exclude the possibility of testicular torsion. In children, color Doppler ultrasound is associated with a sensitivity of 70% and specificity of 88% for epididymitis and a sensitivity of 82% with a specificity of 100% for testicular torsion (61). If testicular torsion is suspected, patients should be immediately referred to a urologist for urgent treatment while waiting for imaging results (62).

Before laboratory analyses are completed, treatment for acute epididymitis should be initiated based on potential pathogens. The goal of early treatment is to cure the infection, alleviate symptoms, prevent transmission, and reduce the risk of future complications. If an infection caused by gonorrhea or chlamydia is suspected, treatment with ceftriaxone and doxycycline is recommended. If there is a potential for E. coli infection or if the patient is allergic to cephalosporins or tetracyclines, treatment should include ofloxacin or levofloxacin. Immunocompromised patients (e.g., those with HIV) can receive the same treatment regimen as immunocompetent patients (54, 63). However, side effects such as allergic reactions, gastrointestinal disturbances, and the development of antibiotic resistance must be considered. In addition to antibiotic treatment, pain relievers, scrotal elevation, activity restriction, and cold compresses can also help to reduce the symptoms of acute epididymitis (63).

#### Chronic epididymitis

Chronic epididymitis is the most common inflammatory condition involving the MRS in clinical practice. While it can result from an acute episode of epididymitis, many patients have had no previous symptoms of acute epididymitis (11). Chronic epididymitis often occurs concomitantly with chronic prostatitis (63).

Symptoms of chronic epididymitis are remarkably varied. Patients may experience local discomfort, a feeling of heaviness, and scrotal pain, which can radiate to the lower abdomen and the inner side of the thigh on the ipsilateral side. Physical examination might reveal an enlarged and hardened epididymis on the affected side. In some cases of chronic epididymitis, a simple lump is palpable in the epididymis without other evident signs or symptoms. The demarcation between the epididymis and the testis is distinct. The spermatic cord and vas deferens may be thickened, and the prostate can feel hardened (64).

The diagnosis of chronic epididymitis requires a careful medical history, physical examination, laboratory tests, and radiological investigations. However, the determination of the exact etiology of chronic epididymitis is usually a challenge because it may be associated with infections, immune reactions, neuropathic pain, or other factors (65). Therefore, the treatment of chronic epididymitis is generally less effective due to unclear etiologies. Treatment approaches for chronic epididymitis include antibiotics, anti-inflammatory drugs, symptomatic treatment, and lifestyle modifications. The antimicrobial agents used in the treatment of chronic epididymitis may not always yield satisfactory outcomes. Long-term use of anti-inflammatory drugs may lead to gastrointestinal problems, kidney damage, and an increased risk of cardiovascular issues. r example, if the patient also suffers from chronic prostatitis, concurrent treatment should be applied, whereas for those with recurrent episodes, epididymectomy may also be an option (66).

## Seminal vesiculitis

### Histology and immune environment of the seminal vesicles

**Figure 4:**
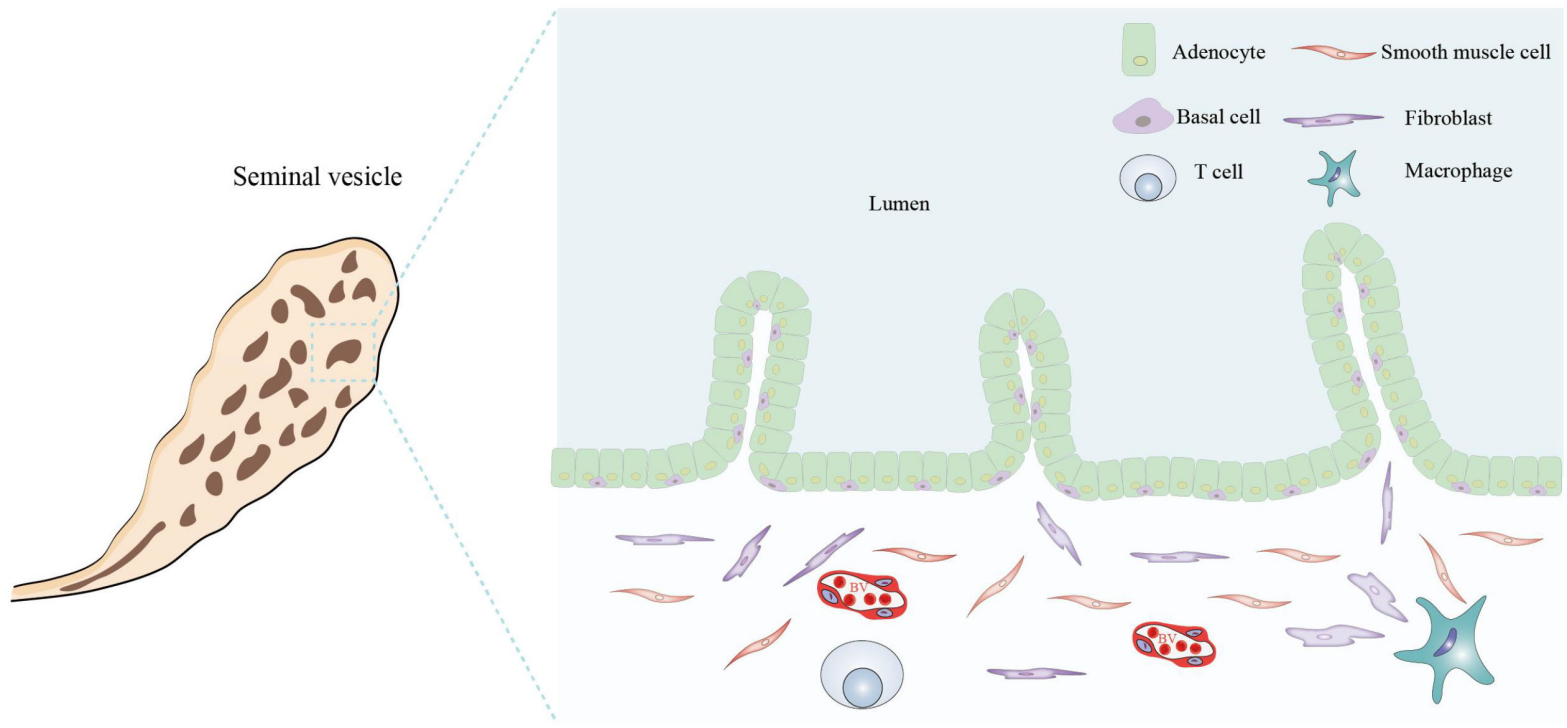
Histological structure and cellular components of the seminal vesicle. The seminal vesicle consists of a coiled tube with a blind end and contains several irregular vesicles within. It is primarily composed of a mucosal layer and a smooth muscle layer. The mucosal surface features non-ciliated, pseudostratified columnar epithelium, which includes glandular cells (adenocytes) and basal cells. The smooth muscle layer predominantly comprises smooth muscle cells, fibroblasts, macrophages, T cells, and BVs. All figures were drawn using Adobe Illustrator software.

The SVs are a pair of spindle-shaped glands composed mainly of SV epithelial cells (SVECs) and minor stromal cells (**Figure 4**). The human SVs are situated at the base of the prostate and between the base of the bladder and the rectum. During ejaculation, the SVs excrete SV fluids to mix with sperm, and the mixed fluids subsequently pass through the prostate and combine with prostatic fluids to form complete semen. SVECs are responsible for the secretion of SVs fluids and may also play a role in the defense against microbial infections through the activation of PRRs (67). Upon exposure to pathogenic microorganisms (e.g., chlamydia) and non-infectious stimuli (such as stones and sperm), the upregulated expression of PRRs in SVECs and the augmented secretion of inflammatory cytokines become significant pathologic mechanisms that induce seminal vesiculitis (68, 69).

### Epidemiology

The seminal vesicles contribute ~70% of semen volume and play a critical role in maintaining sperm viability and vigor (70); and seminal vesiculitis can manifest in men across various age groups. There has been no definitive report on its specific incidence rate, and the prevalence of vesiculitis chiefly hinges on living standards and lifestyles (71). Seminal vesiculitis is not an isolated disease but is highly correlated with chronic prostatitis and epididymitis. Research suggests that the pathways and etiologies of infection for seminal vesiculitis largely mirror those of prostatitis, with both conditions often occurring concomitantly (72).

The seminal vesiculitis can be categorized into acute and chronic disease. Patients with acute seminal vesiculitis may experience high fever, seminal stasis, swelling, and pain (73); and those with chronic seminal vesiculitis commonly manifest hematospermia and/or pain during ejaculation. When coexisting with inflammation of the prostate, symptoms may also include voiding dysfunctions such as frequent urination, urgency, hesitancy, and difficulty in urination (74). Seminal vesiculitis may decrease male semen quality and result in male infertility in severe cases (75, 76). However, hematospermia is a primary manifestation of both acute and chronic seminal vesiculitis, which may severely affect quality of life (77).

### Pathologic mechanisms

While the simultaneous occurrence of seminal vesiculitis with inflammation of other organs of the MRS suggests potential cross-infection, the precise underlying mechanisms remain elusive. The inflammatory lesions of seminal vesiculitis can be coincident with prostatitis and urethritis (72). E.coli and Staphylococcus are common pathogens of infectious seminal vesiculitis (78), and various non-infectious factors such as stones, ejaculatory duct obstruction, and cysts (79, 80)are pivotal in causing non-infectious seminal vesiculitis.

### Diagnostic and therapeutic approaches

The diagnosis of seminal vesiculitis primarily relies on clinical symptoms, radiological examinations, and laboratory tests. For acute seminal vesiculitis, aggressive anti-infection therapy is necessary; and a surgical approach is imperative if SV cysts accompany the condition (81). Chronic seminal vesiculitis is more prevalent than acute cases, in which hematospermia is the primary basis for clinical diagnosis. For patients with persistent hematospermia that does not respond to antibiotic treatment, lifestyle modifications, and vesiculoscopy are effective therapeutic strategies (82). Transrectal ultrasound-guided vesiculoscopy can eliminate potential obstruction and improve vesicular drainage, which can effectively cure hematospermia (83, 84).

## Prostatitis

### Prostate tissue structure and immune environment

**Figure 5:**
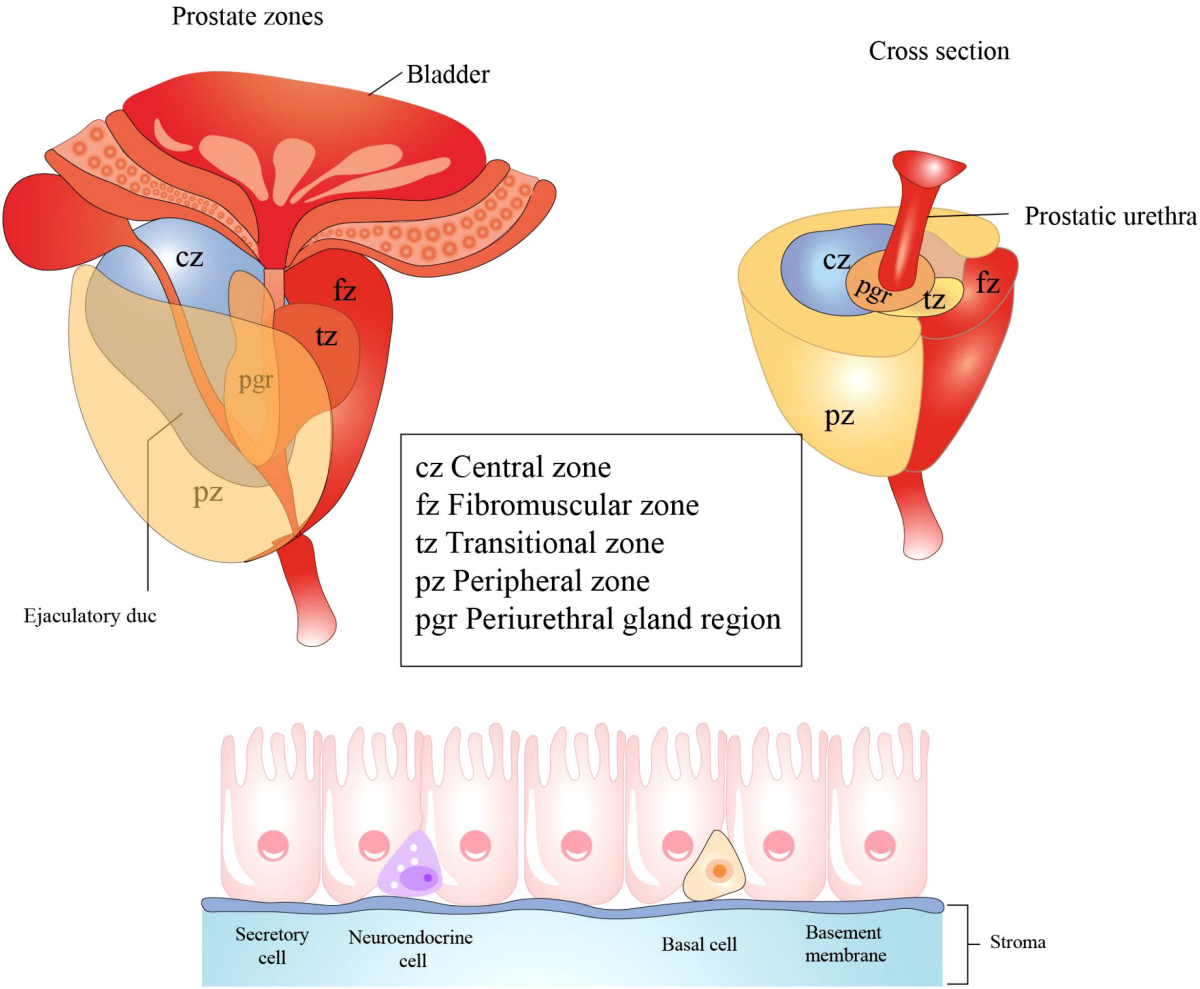
Histological structure and cellular components of the prostate. The prostate primarily consists of three regions: the central zone, situated around the ejaculatory ducts; the transition zone, encircling the urethra; the peripheral zone, which is the largest region. Prostatic epithelial cells play a crucial role in defending against microbial infections of the prostate by secreting antibacterial and antiviral substances. These cells predominantly comprise three cell types: secretory cells, basal cells, and neuroendocrine cells. All figures were drawn using Adobe Illustrator software.

The prostate gland surrounds the initial segment of the urethra between the bladder and the urogenital diaphragm (**Figure 5**). It is composed of three regions: the peripheral and central zones that comprise 95% of the glandular tissue, and the transitional zone that accounts for 5% of the prostate gland (85). The prostate is enveloped by a capsule composed of connective tissue and smooth muscle cells. The prostatic capsule can then be subdivided into three layers: the surface vascular layer, the middle fibrous layer, and the muscular inner layer.

The epithelial cells of the prostate secrete antibacterial and antiviral substances such as defensins and protease inhibitors that play roles in the defense against microbial infection of the prostate (86). However, the prostate is an androgen-dependent organ, and androgens have immunosuppressive properties that can reduce inflammatory responses. Paradoxically, castration or the removal of androgens has also been shown to significantly reduce inflammation in response to microbial infections (87). Various immune cells—including T cells, B cells, macrophages, and mast cells—exist within the prostate and are involved in immune reactions. Due to its anatomic structure and immune microenvironment, the prostate is susceptible to infectious and non-infectious inflammation.

### Epidemiology

Prostatitis is one of the most pathologic conditions in the MRS, predominantly affecting adult males. Its hallmark symptoms include voiding dysfunction and pain in the pelvic and genitourinary tracts, and these may significantly affect quality of life.

The reported prevalence of prostatitis varies widely in the literature due to differences in epidemiological research methods, population surveys, and diagnostic criteria. A community-based cohort study between 1992 and 1996 in Minnesota by Roberts et al. showed that the overall prevalence of diagnosed prostatitis was 9% (88). Similarly, a prevalence of 3.1% and 4.5% was reported in Japan (89). In contrast, the lifetime prevalence of prostatitis is 14.2% in Finland (90). The great variations in the prevalence in different studies may be attributed to differences in diagnostic criteria. Due to the lack of a definitive “gold standard” for the diagnosis of prostatitis, a comparison of its prevalence across various regions is challenging.

### Pathologic mechanisms

The pathogenesis of prostatitis remains largely elusive, and a combination of several factors may contribute to its development. Current research on the etiologic mechanisms of prostatitis primarily focuses on microbial infection, inflammation, abnormal neuromuscular activity of the pelvic floor, immune responses, psychological factors, and neuroendocrine abnormalities. The specific pathomechanisms underlying prostatitis depend upon the distinct etiologies of the disease.

### Bacterial prostatitis

Bacterial prostatitis comprises acute prostatitis and chronic prostatitis, with both conditions primarily caused by bacterial infections. The prostate gland possesses several innate defense mechanisms against infections, including the production of antimicrobial substances and the flushing action of urination and ejaculation on the prostatic urethra (91). However, the impairment of secretory drainage in the prostatic ducts and urine reflux into the prostate can be etiologic factors in inflammation, fibrosis, and calcification. Bacterial prostatitis is most commonly caused by urinary tract infections, with uropathogens possessing specific virulence factors (92); these pathogens are predominantly gram-negative bacteria such as E. coli (93). Risk factors for prostatic infection can be urological instrumentation, urethral stricture, and sexual transmission. Acute bacterial prostatitis is generally more prevalent than chronic bacterial prostatitis (94).

### Chronic non-bacterial prostatitis

Chronic non-bacterial prostatitis represents the most common inflammatory condition in the prostate and manifests complicated etiologies. The specific etiologic factors of most chronic non-bacterial prostatitis are largely unclear, and it is uncertain as to whether a microbial infection is related to chronic non-bacterial prostatitis. Some studies suggest that covert bacterial infection of the prostate might be responsible for some cases of chronic prostatitis (95). One study from Korea suggests that chronic non-bacterial prostatitis is derived from bacterial infection (96). However, substantial evidence for the correlation between chronic non-bacterial prostatitis and bacterial infection is largely missing. Although reflux of urine (primarily uric acid) into the prostate is speculated to be one of the etiologic factors of chronic non-bacterial prostatiti (97), this hypothesis is still debated.

Chronic non-bacterial prostatitis may be linked to autoimmunity, as the immune response to prostate-specific antigen (PSA) may lead to chronic inflammation. Evidence supporting this hypothesis includes the presence of autoantibodies against PSA and T-cell responses in chronic prostatitis patients (98).

Elevated levels of pro-inflammatory cytokines—principally interleukin (IL)-1, IL-6, and tumor necrosis factor-alpha (TNF-α)—have also been observed in prostatic fluids of chronic prostatitis patients and are associated with inflammatory conditions (99).

Psychological factors and neuroendocrine mechanisms are emerging as underlying factors in chronic prostatitis. Dysfunctions in peripheral and central pain regulation are correlated with chronic pelvic pain in prostatitis patients. Persistent inflammation in the prostate may enhance neuronal sensitivity, thereby resulting in magnification and prolongation of pelvic pain. Psychological factors such as stress and anxiety may also amplify pain symptoms of chronic prostatitis. However, whether such psychological factors constitute a direct etiology or a secondary effect remains unclear (100).

### Diagnosis and classification

Prostatitis presents with diverse symptoms, such as lower urinary tract dysfunction, sexual impairment, and altered semen parameters (101)and may severely affect male health. Since >90% of chronic prostatitis cases lack an infectious etiology (102), an empirical diagnosis beyond laboratory tests is important. Therefore, the diagnosis of chronic prostatitis is challenging, and misdiagnosis occurs frequently. Although clinicians have developed various diagnostic methods, these remain disputed within the medical community.

The Meares-Stamey “four-glass test” was the first classification standard for prostatitis (103). By examining the number of white blood cells and bacterial culture results in an initial urine sample (voided bladder one, VB1), mid-stream urine sample (voided bladder two, VB2), expressed prostatic excretion (EPS), and post-prostatic massage urine sample (voided bladder three, VB3), prostatitis was classified into acute bacterial prostatitis, chronic bacterial prostatitis, chronic non-bacterial prostatitis, and prostatodynia. Due to the complexity of the “four-glass test” and challenges in obtaining prostatic fluid, a “two-glass test” has been developed, in which leukocytes and bacteria are examined in the urine (104). However, the correlations between leukocyte or bacterial counts and prostatitis symptoms are not always consistent (105); thus, neither the “four-glass” nor “two-glass” test is useful in establishing a definitive diagnosis of prostatitis.

In the 1990s, the United States National Institutes of Health (NIH) established a classification system for prostatitis (106). Based on this diagnostic system, only a minority of prostatitis cases are defined as bacterial infections, including acute bacterial prostatitis (type I) or chronic bacterial prostatitis (type II). Greater than 90% of cases are defined as non-bacterial chronic prostatitis/chronic pelvic pain syndrome (CP/CPPS, type III) (101). Type IIIA, also known as inflammatory CP/CPPS, is characterized by symptoms of prostatitis associated with an increased white blood cell count in semen or prostatic fluids. In Type IIIB or noninflammatory CP/CPPS, there is no laboratory evidence of inflammation. Finally, type IV prostatitis is defined as reflecting a lack of clinical symptoms but with evidence of inflammation found in laboratory examinations of EPS, semen, and/or prostatic biopsies.

### Therapeutic approaches

Due to the absence of unified diagnostic criteria, the therapeutic approach to prostatitis varies.

### Antibiotics

Antibiotics comprise an effective treatment for type I and type II prostatitis, as acute bacterial prostatitis is sensitive to antibiotic treatment. The choice of antibiotics should be based on bacterial culture and sensitivity results, and patients with risk factors for antibiotic resistance require intravenous treatment with a broad-spectrum antibiotic regimen (107). The duration of antibiotic treatment is 2 weeks for patients with mild infections and may be 4 weeks for patients with severe symptoms (108). While antibiotic treatment is ineffective for non-infectious prostatitis, it may be used when a diagnosis is unclear.

### α-Adrenergic blockers

α-Adrenergic blocking agents relax smooth muscle in the prostate and bladder neck, thereby improving lower urinary tract symptoms and pelvic pain in patients with prostatitis. Hence, these drugs are frequently used to treat type II/type III prostatitis. The combination of α-blockers with antibiotics is also more effective than antibiotics alone (109). In addition, α-blockers have side effects such as first-dose syncope, dizziness, tachycardia, hypotension, headache, asthenia, rhinitis, and ejaculatory dysfunction, which should be considered when prescribing these medications (110).

### Nonsteroidal anti-inflammatory drugs

NSAIDs, mainly rofecoxib and celecoxib, are frequently used to treat CP/CPPS and can significantly reduce pain symptoms (111). However, the potential adverse effects of NSAIDs must be considered on a case-by-case basis. NSAIDs are useful for managing inflammation but have potential long-term side effects, including kidney impairment, gastrointestinal bleeding, and increased cardiovascular risks (112).

### Non-drug treatments

In addition to the aforementioned medications, some patients with prostatitis may benefit from treatment with shock waves, surgical procedures, and acupuncture (113–115). Due to the complex etiology and diverse symptoms of prostatitis, a combination of several therapeutic modalities may give rise to improved outcomes.

### Sperm immunogenicity and inflammation

In mammals, sperm are initially produced after puberty, years after central immune tolerance has been established during the fetal and neonatal periods. Sperm do not induce immune responses in the testes and epididymis under physiologic conditions because these organs adopt immune-privileged environments (116). However, sperm may induce autoimmune responses under certain pathologic conditions (117). More than 70% of men who have undergone vasectomy produce anti-sperm antibodies (118), which is attributed to sperm release into the abdominal cavity due to vasectomy (119).As immunogenic agents, sperm also induce non-infectious inflammation in the MRS under certain pathologic conditions.

Recent studies using mouse models have shown that sperm can induce inflammation in the MRS through several mechanisms.

① *In vitro* experiments demonstrate that damaged spermatogenic cells stimulate Sertoli cells to express various inflammatory mediators, including TNF-α, IL-1β, IL-6, and monocyte chemoattractant protein-1 (MCP-1) through the activation of TLR2 or TLR4 (120). Additionally, damage to spermatogenic cells elevates the expression of TNF-α and MCP-1 in the testis and promotes immune cell infiltration. These observations suggest that spermatogenic cell damage may trigger autoimmune testicular inflammation by inducing the expression of inflammatory genes in Sertoli cells.

②Sperm damage induces non-infectious epididymitis (121). After peritoneal injection of busulfan in mice, sperm are damaged and accumulate in the epididymis, and, accordingly, the epididymitis characterized by immune cell infiltration becomes evident. Sperm damage induces the expression of inflammatory cytokines such as TNF-α, IL-6, and IL-1β, as well as MCP-1, MCP-5, and C-X-C motif chemokine 10 (CXCL10) in the epididymal epithelial cells; and these agents are responsible for infiltration into the epididymis.

③ Sperm cells induce vesiculitis in mice (69). When sperm were locally injected into the SV, swelling was observed in the mouse SV, accompanied by evidence of leukocyte infiltration. Sperm significantly increased the expression of major inflammatory cytokines, including TNF-α, IL-6, MCP1, and CXCL10 in SVECs; and NF-κB was activated in SVECs after sperm challenge, which is essential for the expression of inflammatory cytokines. These results indicate that sperm may induce non-infectious vesiculitis through the activation of an innate immune response in the SVECs (69). In accordance with these results in mice, sperm are frequently found in the fluids of the SVs from patients.

Urinary reflux into the prostate is a significant cause of non-bacterial prostatitis and leads to leukocyte infiltration in the stroma of the rat prostate and a coincident increase in the expression of IL-1α, IL-1β, IL-6, and TNF-α (97). Although direct evidence of sperm-induced prostatitis is missing, it is reasonable to speculate that immunogenic sperm could induce non-infectious prostatitis if they are refluxed into the prostate.

Based on the aforementioned observations and speculations, one can hypothesize that structural disorders or obstruction in the genital tracts under some pathologic conditions may lead to sperm damage or reflux into genital accessory glands, including the prostate and SVs where sperm are not present under normal physiologic conditions. The immunogenicity of sperm may thus result in inflammatory conditions in these accessory glands (which are immunocompetent). Clarification of this hypothesis is important since it can provide insights into the etiologic factors of non-infectious inflammation in the MRS, which can then aid in the development of preventive and therapeutic approaches to the aforementioned disorders of the MRS.

## Conclusions

In the MRS, inflammatory conditions are common and can significantly impair quality of life and male fertility. The etiology and mechanisms underlying these conditions, however, remain incompletely understood. The MRS, a relatively isolated entity, comprises several organs that are each enclosed within distinct environments. These organs have unique histological and immunological characteristics crucial for their functions and are implicated in MRS inflammation. Numerous critical questions regarding MRS inflammation require urgent attention. For example, the optimal timing for therapeutic intervention in MuV infection to preserve spermatogenic function and hinder disease progression remains unclear. Additionally, there is a lack of robust, evidence-based medical data to support specific treatment strategies. Chronic low-grade inflammatory responses, such as chronic prostatitis and epididymitis, may affect spermatogenesis, but the exact mechanisms are yet to be elucidated. This necessitates further research into the pathogenesis of these conditions and the development of more effective treatments.

While acute inflammatory conditions in the MRS are often treated as infectious diseases with antibiotics, there is usually a lack of solid evidence of microbial infection in chronic inflammation. The pathogens responsible for chronic inflammation in the MRS and their mechanisms of action need urgent clarification to improve diagnostic and treatment strategies. Moreover, the role of sperm in chronic MRS inflammation is often overlooked, despite their immunogenic nature and prolonged presence in the MRS.

In addition, addressing specific research gaps is crucial for advancing understanding and treatment in MRS inflammation. Future studies should focus on elucidating the mechanisms of chronic non-infectious inflammation, particularly the role of immune cells, cytokines, and molecular signaling pathways. Research on the long-term effects of orchitis and epididymitis on male fertility is also necessary, with longitudinal cohort studies assessing the impact of different treatments. Investigating the role of sperm immunogenicity in chronic inflammation and developing novel immunomodulatory therapies targeting specific immune pathways will be important for improving therapeutic outcomes. Moreover, exploring personalized treatment strategies based on patient-specific factors could enhance treatment efficacy and patient care. Addressing these gaps will provide valuable insights into the mechanisms of MRS inflammation and lead to more effective treatments.

Overall, a deeper understanding of the etiologic factors and mechanisms driving chronic non-infectious inflammation in the MRS is required to meet clinical needs in diagnosis and treatment.
