# Correction: Inflammation of the male reproductive system: clinical aspects and mechanisms

**DOI:** 10.3389/fendo.2025.1649509

**Published:** 2025-07-02

**Authors:** Binghao Bao, Haolang Wen, Fei Wang, Daishu Han, Baoxing Liu

**Affiliations:** ^1^ Department of Andrology, China-Japan Friendship Hospital, Beijing, China; ^2^ Graduate School, Beijing University of Chinese Medicine, Beijing, China; ^3^ Institute of Basic Medical Sciences, Chinese Academy of Medical Sciences, School of Basic Medicine, Peking Union Medical College, Beijing, China

**Keywords:** male reproductive system, inflammation, spermatozoa, diagnosis, treatment

In the published article, there was an error in **Figure 3**, **Figure 4** and **Figure 5** as published. **Figure 3**, **Figure 4** and **Figure 5** are positioned incorrectly in the published version. The figures have been swapped, although the figure captions remain correct and in their proper positions. The corrected **Figure 3**, **Figure 4** and **Figure 5** and its caption appear below.

**Figure 3:**
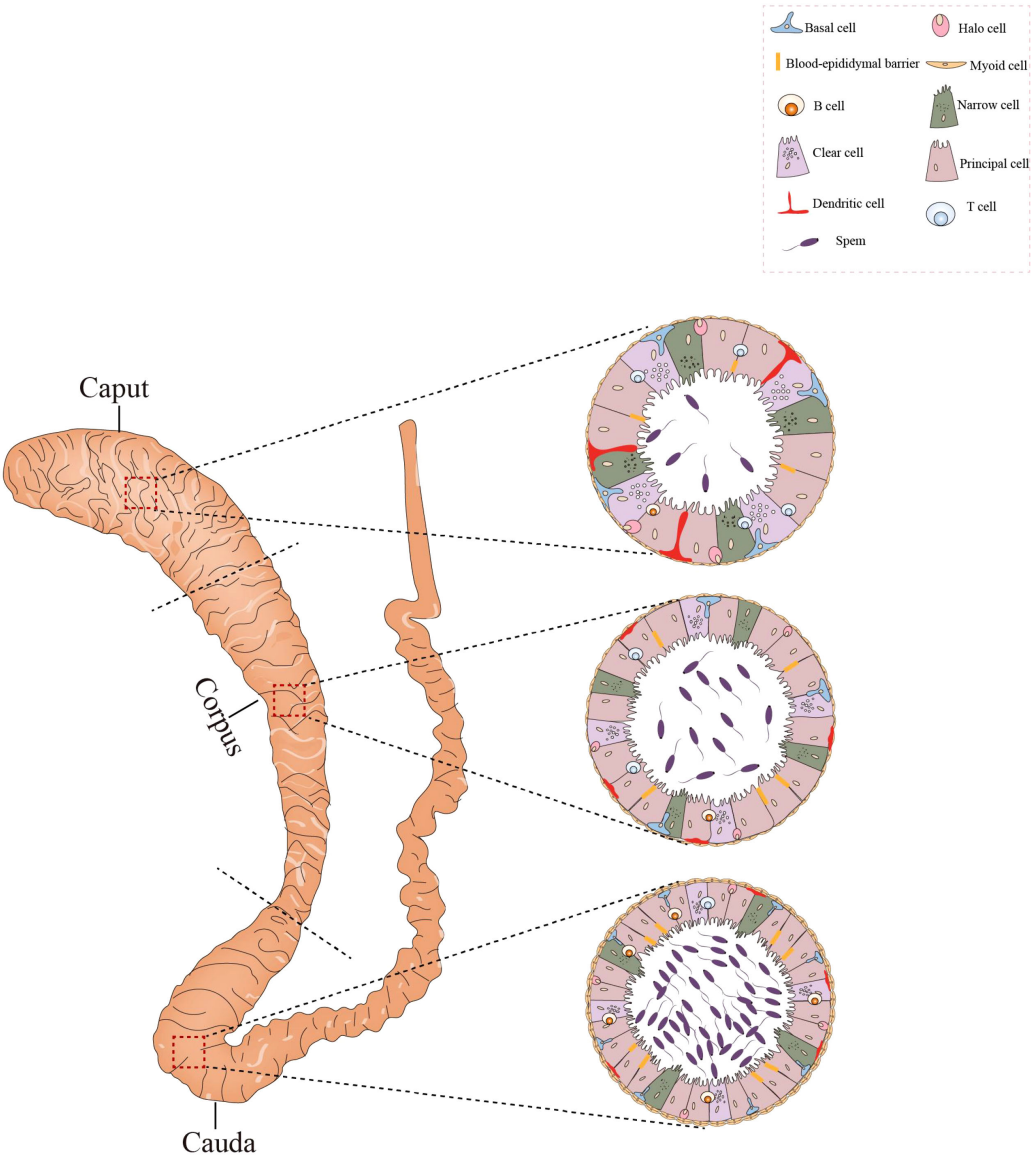
Histological structure and cellular components of the epididymis. The epididymis consists of three sections: the caput, corpus, and cauda. Its epithelium harbors diverse cell types, such as basal cells, lymphocytes, clear cells, dendritic cells, and macrophages. In the caput, dendritic cells can extend towards the tight junctions between epithelial cells. The caput contains a higher number of T cells compared to the cauda, whereas the concentration of B cells is greater in the cauda. All figures were drawn using Adobe Illustrator software.

**Figure 4:**
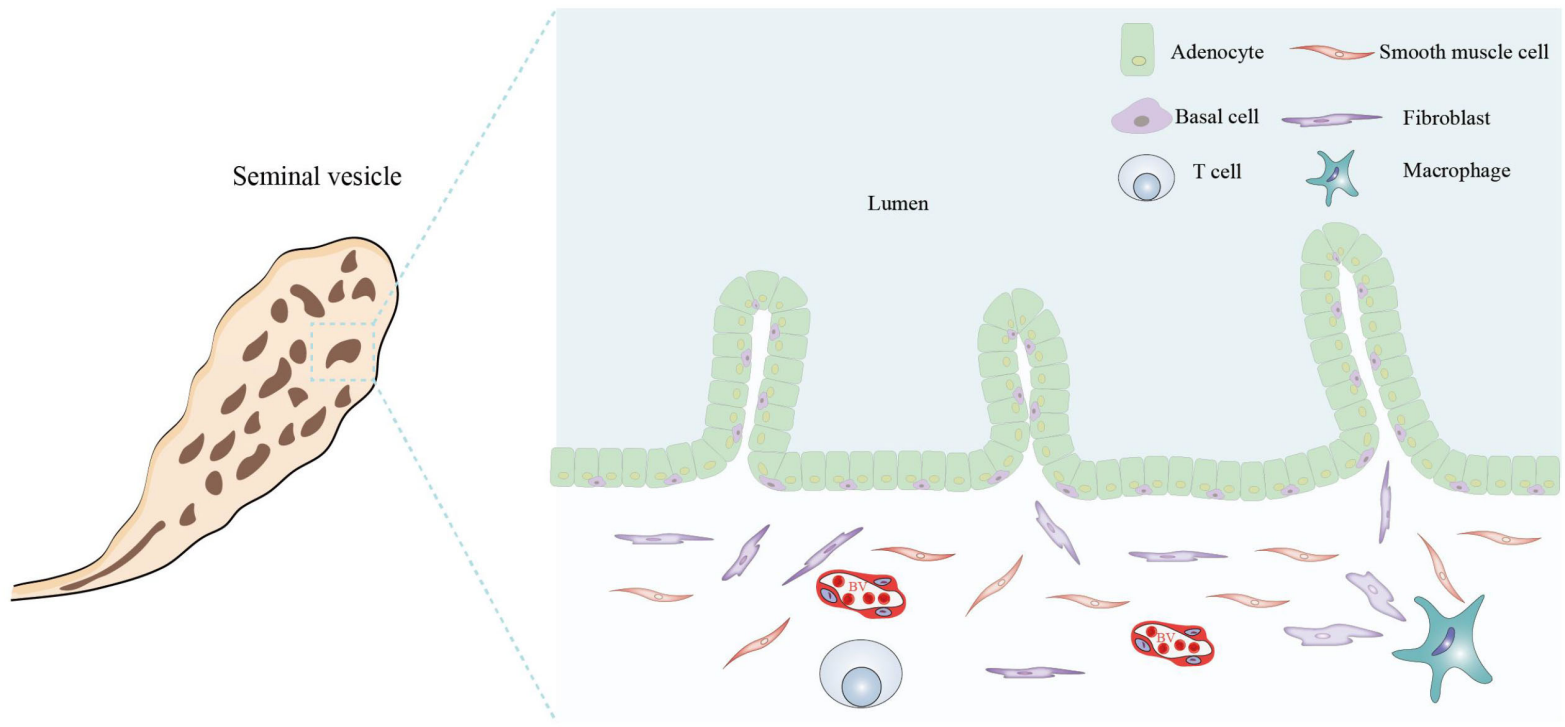
Histological structure and cellular components of the seminal vesicle. The seminal vesicle consists of a coiled tube with a blind end and contains several irregular vesicles within. It is primarily composed of a mucosal layer and a smooth muscle layer. The mucosal surface features non-ciliated, pseudostratified columnar epithelium, which includes glandular cells (adenocytes) and basal cells. The smooth muscle layer predominantly comprises smooth muscle cells, fibroblasts, macrophages, T cells, and BVs. All figures were drawn using Adobe Illustrator software.

**Figure 5:**
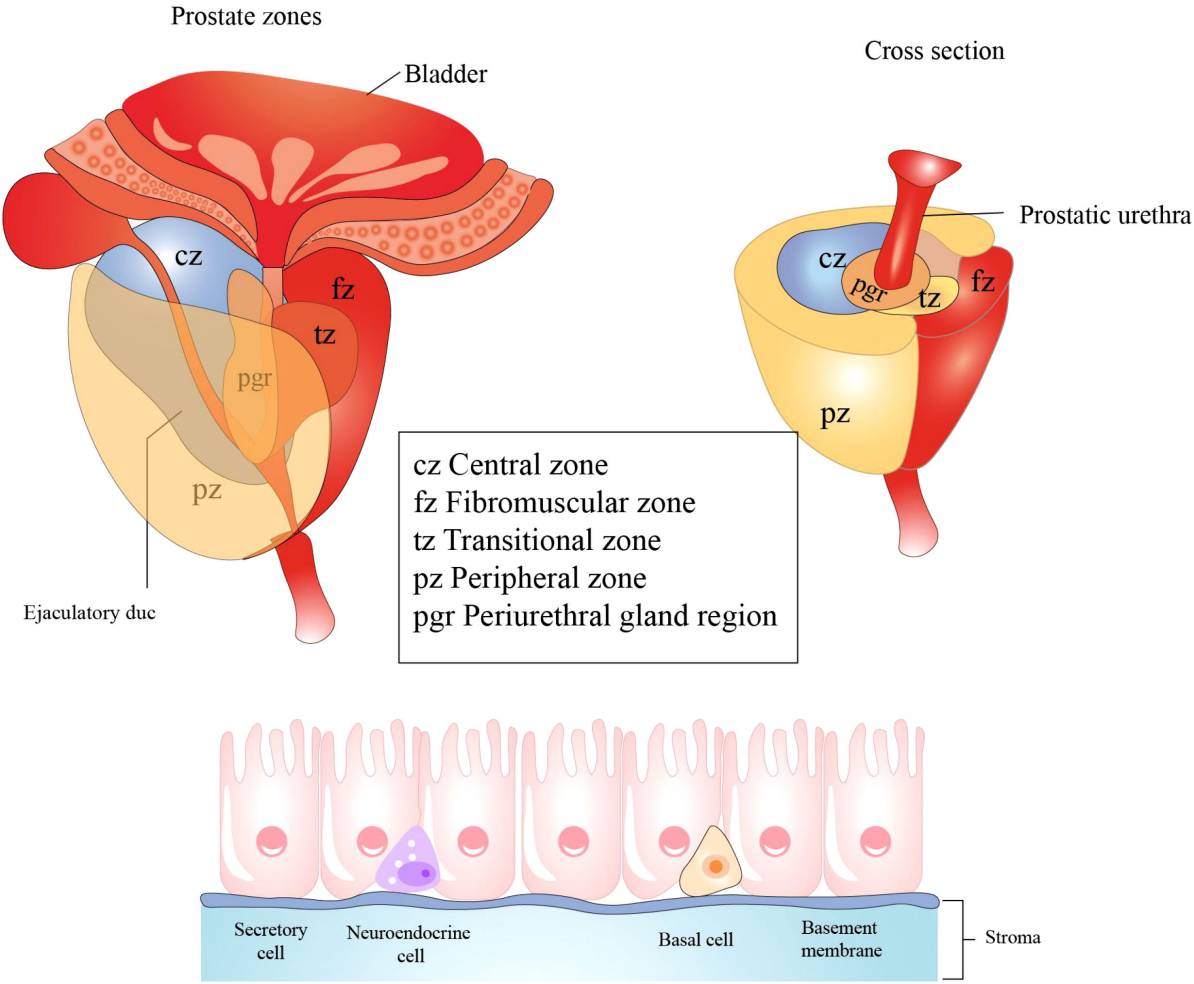
Histological structure and cellular components of the prostate. The prostate primarily consists of three regions: the central zone, situated around the ejaculatory ducts; the transition zone, encircling the urethra; the peripheral zone, which is the largest region. Prostatic epithelial cells play a crucial role in defending against microbial infections of the prostate by secreting antibacterial and antiviral substances. These cells predominantly comprise three cell types: secretory cells, basal cells, and neuroendocrine cells. All figures were drawn using Adobe Illustrator software.

The authors apologize for this error and state that this does not change the scientific conclusions of the article in any way. The original article has been updated.

